# Faith in frames: unveiling therapeutic narratives in religion-related cinema through computational analysis

**DOI:** 10.3389/fpubh.2024.1385379

**Published:** 2024-05-20

**Authors:** Bai Xue, Zhongrui Wang, Yuqing Liu, Yao Song

**Affiliations:** ^1^College of Humanities and Social Sciences, Xi’an Jiaotong University, Xi’an, China; ^2^Convergence Laboratory of Chinese Cultural Inheritance and Global Communication, Sichuan University, Chengdu, China; ^3^College of Literature and Journalism, Sichuan University, Chengdu, China

**Keywords:** religion, films, narrative, cinema therapy, emotions

## Abstract

**Introduction:**

This study explores the emotional impact of religion-related films through a “cinematherapy” lens. It aims to analyze the emotional patterns in a curated selection of religion-related films compared to a broader sample of acclaimed movies using facial recognition with YOLOv5 object detection. The study aims to uncover the potential therapeutic application of religion-related films.

**Methods:**

Facial recognition with YOLOv5 object detection was utilized in this study to analyze the emotional patterns in religion-related films. A curated selection of these films was compared to a broader sample of acclaimed movies to identify any distinct emotional trajectories.

**Results:**

The analysis of the emotional patterns revealed that religion-related films exhibited a subtler range of emotions compared to the broader film spectrum. This finding suggests that these films potentially create a safe space for contemplation, aligning with the profound themes often explored in religion-related films. Interestingly, the emotional arc observed in the films mirrored the spiritual journeys depicted in them. The films started with a low point of separation, transitioned through challenges, and culminated in a peak representing spiritual transformation.

**Discussion:**

These findings suggest promise for the therapeutic application of religion-related films. The muted emotional expression in these films creates a safe space for self-reflection, enabling viewers to connect with the struggles of the characters and explore their own values when faced with complex religious ideas. This emotional engagement may contribute to therapeutic goals such as introspection and personal growth. The study unveils the unique emotional power of religion-related films and paves the way for further research on their potential as therapeutic tools. It emphasizes the need for continued exploration of the emotional impact of these films and their capacity to aid in therapeutic goals.

## 1 Introduction

Film has captivated audiences since its inception, weaving narratives that transport us to different worlds, evoke a range of emotions, and challenge our perspectives (1). Cinematic theory delves deeper, analyzing the language of film itself – its use of camera angles, editing techniques, and sound design to create meaning and impact (2). Research has extensively explored film’s power to influence our thoughts, beliefs, and behaviors (3). Indeed, films can spark social movements, promote empathy, and even provide a form of therapy, known as “Cinematherapy “(4). For instance, documentaries like “An Inconvenient Truth” ignited the climate change movement, while powerful dramas like “Schindler’s List” fostered empathy for the horrors of the Holocaust. Cinematherapy utilizes films to address various psychological issues (5). Studies have shown that movies exploring themes of addiction can be particularly helpful in recovery programs (6, 7).

Religion, another powerful force in human experience, permeates societies and shapes cultures (8). Inevitably, film, as a mirror reflecting society, has become a platform for exploring religious themes and narratives (9). This genre, encompassing films that substantially engage with religious ideas, practices, or symbolism, presents a wide spectrum of religious experiences (10). These films can range from portraying specific religious traditions to exploring broader spiritual and existential questions (11).

Religion-related films, which refers to those that substantially engage with religious themes, narratives, or symbolic representations, transcending specific faiths (11), hold a special place in cinematic storytelling (12). They delve into profound themes of faith, morality, and personal transformation, often through captivating narratives that resonate deeply with audiences (13). These films transcend mere entertainment, offering solace, guidance, and a framework for navigating the complexities of life (12). Their potent combination of spiritual themes and emotional engagement positions them uniquely to influence viewers on a deeper level (14). Religion-related films grapple with profound questions about the meaning of life, the nature of good and evil, and our place in the universe (3). These themes resonate with viewers’ core values and beliefs, creating a powerful emotional connection (15). Furthermore, filmmakers utilize a range of techniques to evoke emotions, such as music, cinematography, and compelling narratives (16). This emotional engagement allows viewers to connect with the characters and their journeys on a personal level, fostering reflection and growth (1).

While research confirms the effectiveness of cinematic therapy, particularly with religion-related films (17, 18), a key question remains unanswered: what specific elements within these films drive this positive impact? Existing studies highlight the films’ ability to provide emotional support, self-reflection, and personal growth (19). For example, films exploring themes of forgiveness, like “Gran Torino,” can offer solace to viewers grappling with similar issues. Documentaries portraying real-life struggles with faith, such as “God Grew Tired of Us,” can encourage introspection and a deeper understanding of one’s own beliefs. However, it’s the skillful crafting of emotional narratives that truly unlocks cinematic therapy’s potential (20). The presentation of moral dilemmas, the depiction of transformative journeys, and the exploration of existential themes all resonate deeply with viewers’ emotions (21). This emotional connection offers solace, guidance, and a framework for navigating life’s challenges (22). Furthermore, the recurring motif of personal transformation may provide viewers with a framework for introspection, inspiring them to explore their potential for growth (5). However, a crucial gap exists in understanding the “why” behind this effectiveness. While the therapeutic impact is recognized, the specific narrative patterns within the films that trigger these positive responses remain largely unexplored.

Our study aims to address this gap by focusing on the power of narrative patterns, particularly emotional patterns, within religion-related films. We hypothesize that the way these films evoke and explore emotions plays a key role in their therapeutic influence. Considering emotional patterns are multifaceted, encompassing both characters’ expressions and the audience’s internal journey (22), we specially use facial expression as a profound canvas to capture a spectrum of experiences and psychological resonance (23). Surpassing cultural and language differences, facial expressions allow viewers from all walks of life to connect with the characters’ emotional experiences (24). Besides, previous research on cinematic therapy has often relied on case studies or small-scale experiments, limiting the generalizability of findings (17, 18). Our study proposes a novel methodology that utilizes computational techniques to overcome this limitation. Tools like sentiment analysis, emotion tracking, and machine learning, were used to analyze the emotional arcs within these films (25). This innovative approach will allow us to quantify and analyze the emotional impact of religious narratives on a larger scale, providing a deeper understanding of how these films shape viewers’ values and emotional landscapes.

## 2 Materials and methods

### 2.1 Sampling

The current research aims to examine the unique emotional patterns present in religion-related films. To achieve these objectives, two distinct sampling approaches were employed.

Firstly, to explore the emotional patterns intrinsic to religion-related films, a curated sample of 25 religion-related films was selected from Stacker, an authoritative data curation platform (26). Stacker compiles lists across various domains, including the top 25 religion-related films as appraised by critics from multiple databases, magazines, and websites (26). These films present a diverse array of religious themes, highlighting various aspects of faith, redemption, sacrifice, and spiritual awakening, thereby fulfilling the criterion of representativeness (26). Since there exists a lack of research utilizing film metrics to examine the propagation of religion-related films in secular contexts, this study aims to address this gap through an in-depth analysis using film metrics methodologies and measurement instruments.

**Figure 1:**
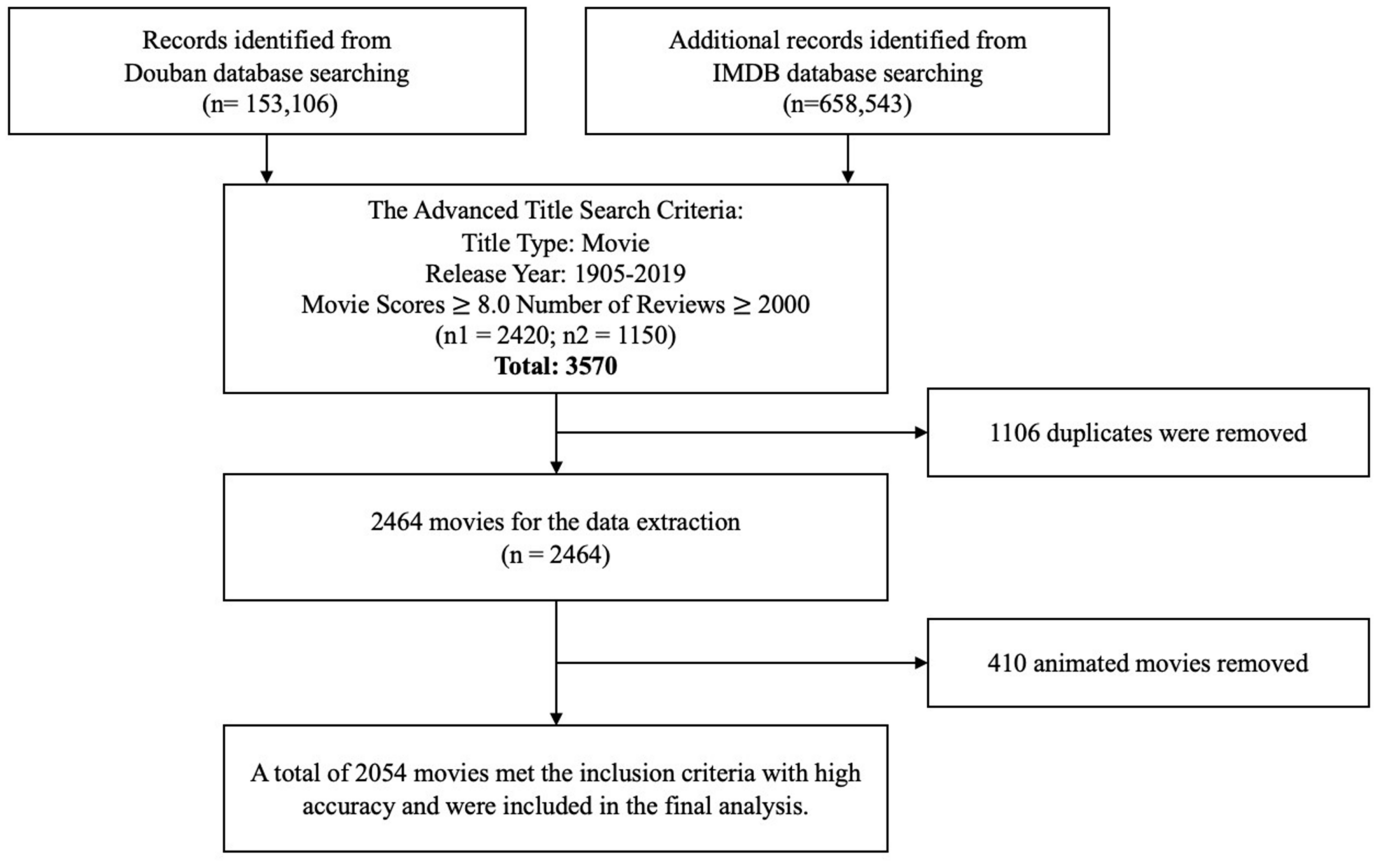
The process diagram for selecting 2054 high-quality films.

Secondly, to discern if the emotional patterns observed in religion-related films are unique or simply reflective of broader cinematic trends, a comparative sample of 2,464 critically acclaimed films from 1905 to 2019 was compiled. Each of these films garnered an impressive rating (8.0 or higher) and substantial viewer feedback (more than 2,000 comments) on the influential platforms Douban and IMDB - hallmarks of mainstream popularity and recognition (27). By contrasting the emotional patterns across the religion-related film sample and this comparative sample of highly acclaimed works from various genres, any distinctiveness specific to religion-related films could be ascertained while accounting for potential confounding factors present in the general film landscape (28). Considering current emotion detection technologies are predominantly trained on datasets consisting of human expressions and emotional values, we excluded movies labeled with “animation” to ensure the accuracy of our results. After these manipulations, we ultimately retained a total of 2054 high-quality films. The entire movie selection process is illustrated in Figure 1.

### 2.2 Measurement

This study prioritizes facial expressions as the core metric for analyzing emotional impact in religion-related films. This choice aligns with the well-established role of emotions in film’s narrative power (29). Every cinematic element contributes to emotional portrayal, but the human face remains a universal and primary canvas for expressing emotions (30, 31). Facial expressions transcend cultural and linguistic barriers, allowing viewers from diverse backgrounds to connect with the characters’ emotional journeys (24). This is particularly relevant for religion-related films, which often explore universal themes of faith, hope, and existential questioning (22). Analyzing facial expressions in these films allows us to capture the emotional core of religious narratives, even when the specific religious iconography or rituals might differ across cultures (32).

Furthermore, focusing on facial expressions aligns with the goals of cinema therapy research, which investigates the potential for films to evoke emotional responses that contribute to psychological well-being (33). Facial expressions are a crucial element in viewers’ emotional engagement with characters (31). By analyzing the emotional trajectories depicted on characters’ faces, we can gain insights into how religion-related films might influence viewers’ own emotional states and potentially provide solace or inspiration.

### 2.3 Technical approach

The crux of this research endeavor lies in accurately capturing and quantifying the emotions portrayed in films. As a prerequisite, recognizing emotions necessitates the identification of characters within the film frames. To accomplish this objective, after a comprehensive evaluation and comparison of cutting-edge technologies, we selected the YOLOv5 visual recognition framework to identify characters across the film frames (34).

**Figure 2:**
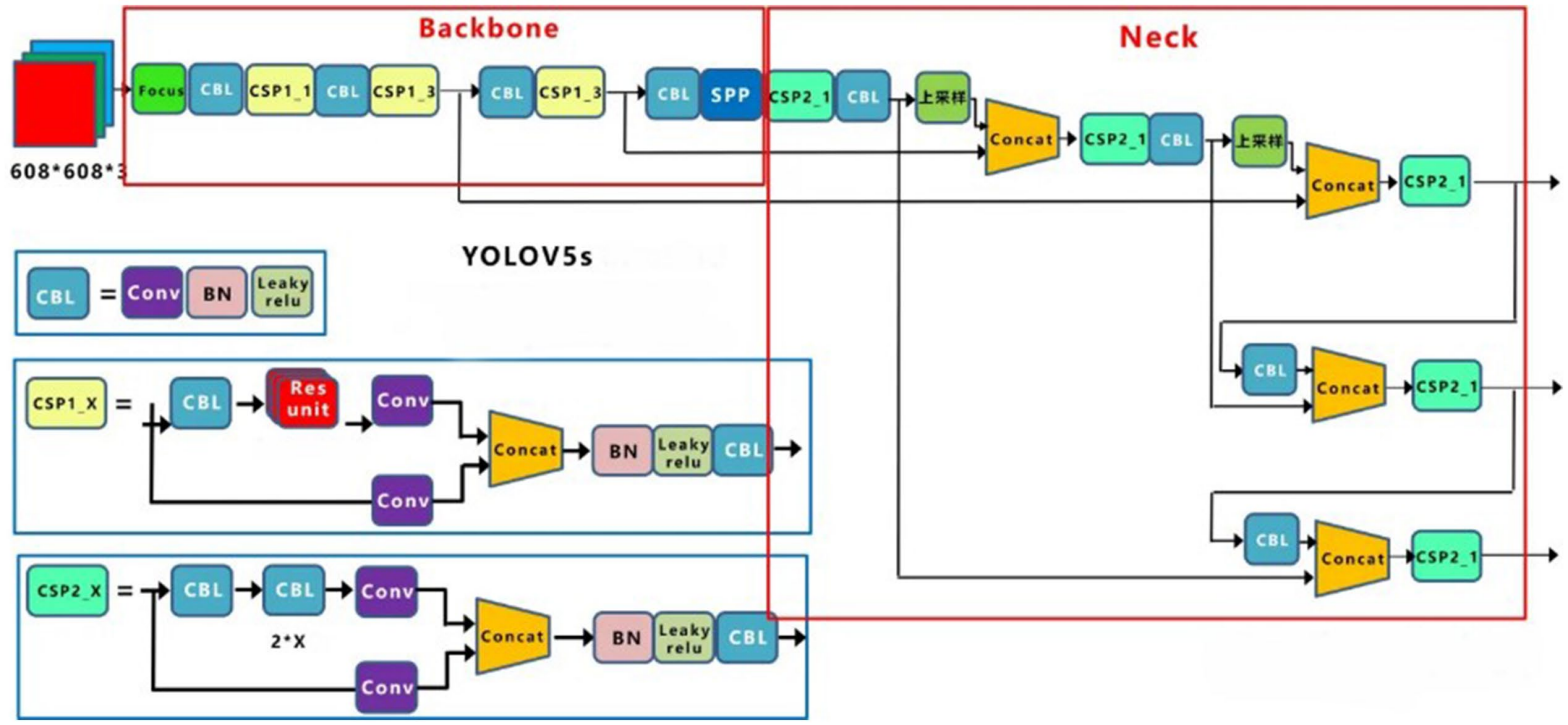
Structure of YOLOv5s.

YOLOv5 is an object detection framework based on deep learning that can achieve fast, accurate real-time object detection (34, 35). It is one member of the YOLO (You Only Look Once) family, and incorporates many cutting-edge technologies to further enhance the performance and efficiency of object detection (35). Compared to other object detection techniques, it is characterized by its lightweight structure, extreme speed, and support for multi-scale inference (36). The model structure of YOLOv5 is depicted in Figure 2.

Considering that movies are typically presented at a rate of 24 frames per second, our team conducted a comprehensive study of shot segmentation across more than 2,000 films using Python. Our findings revealed that 99% of the shots exceeded 0.5 s or 12 frames in duration. To strike an optimal balance between speed and accuracy, we extracted one image from the film for every 12 frames and performed human body identification and localization on each extracted frame. For this purpose, we employed the human body identification model from the COCO dataset to achieve optimal results.

From receiving an image input to outputting the final results, YOLOv5 undergoes a series of processing steps, including data preprocessing, feature extraction, bounding box generation, prediction output, non-maximum suppression, and post-processing. With each new image input, the model uniformly scales the image. Specifically, in the image scaling step, if the original image size is (H x W) and the target size is (h x w), the pixel position after scaling can be calculated using the following formula:


$$\left(i',j'\right)=\left(\frac{i}{H}h,\frac{j}{W}w\right)$$

Where i and j are the pixel positions of the original image, and i’ and j’ are the pixel positions after scaling.

At the final result output stage, the model filters and cleans detection results with low confidence scores, only outputting predicted recognition results that meet a certain threshold. The filtering logic can be represented as follows: $B_{\mathrm{final}}=B_{\mathrm{pred}}\mathrm{| score}\left(B_{\mathrm{pred}}\right)>\mathrm{threshold}$ Where $B_{\mathrm{final}}$ is the final detection result, $B_{\mathrm{pred}}$ is the predicted bounding box, $\mathrm{score}\left(B_{\mathrm{pred}}\right)$ is the confidence score of the bounding box, and threshold is the set threshold.

After capturing all human figures in the film, emotion recognition is performed to analyze the local facial expressions while considering the background context (37), which is particularly well-suited for the scenario of emotion recognition in films.

**Figure 3:**
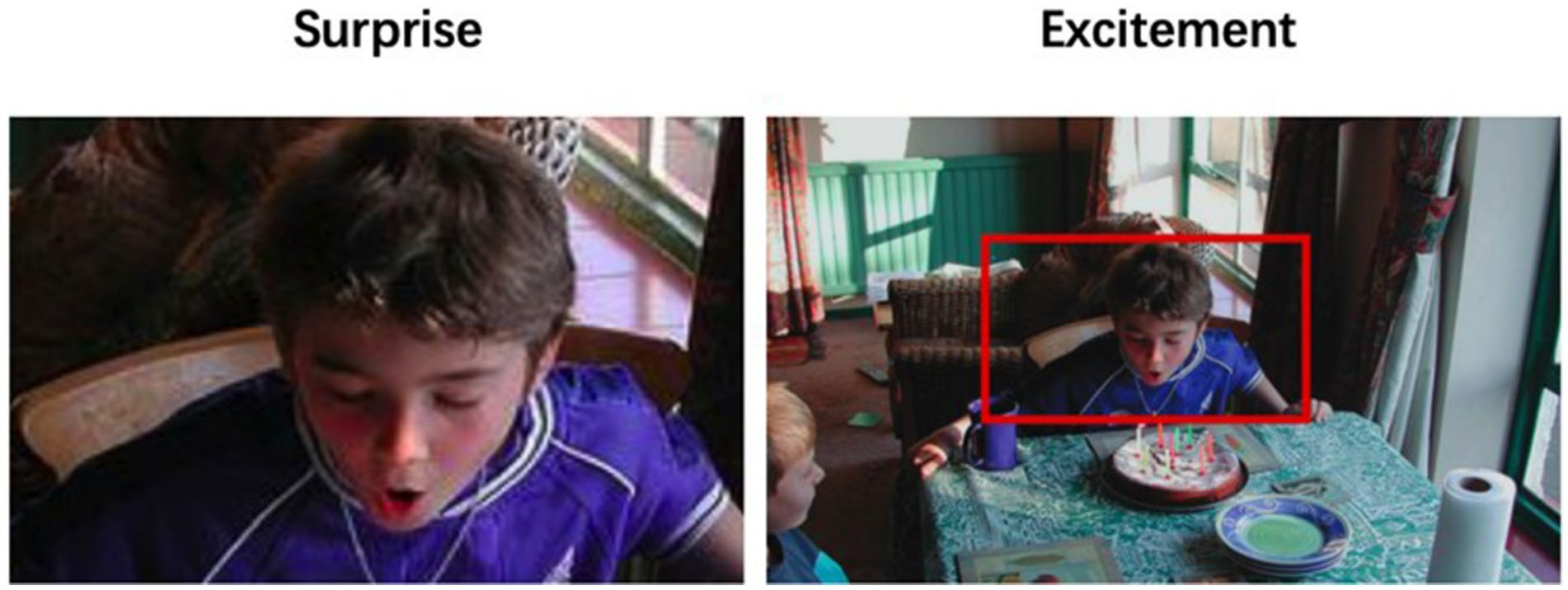
Two types of emotion recognition (37).

For example, in Figure 3, if one were to analyze the emotion of the little boy in the picture in isolation, the most probable interpretation would be surprise. However, when considering the context of the boy’s birthday celebration, happiness and excitement become more accurate representations of his emotional state.

**Figure 4:**
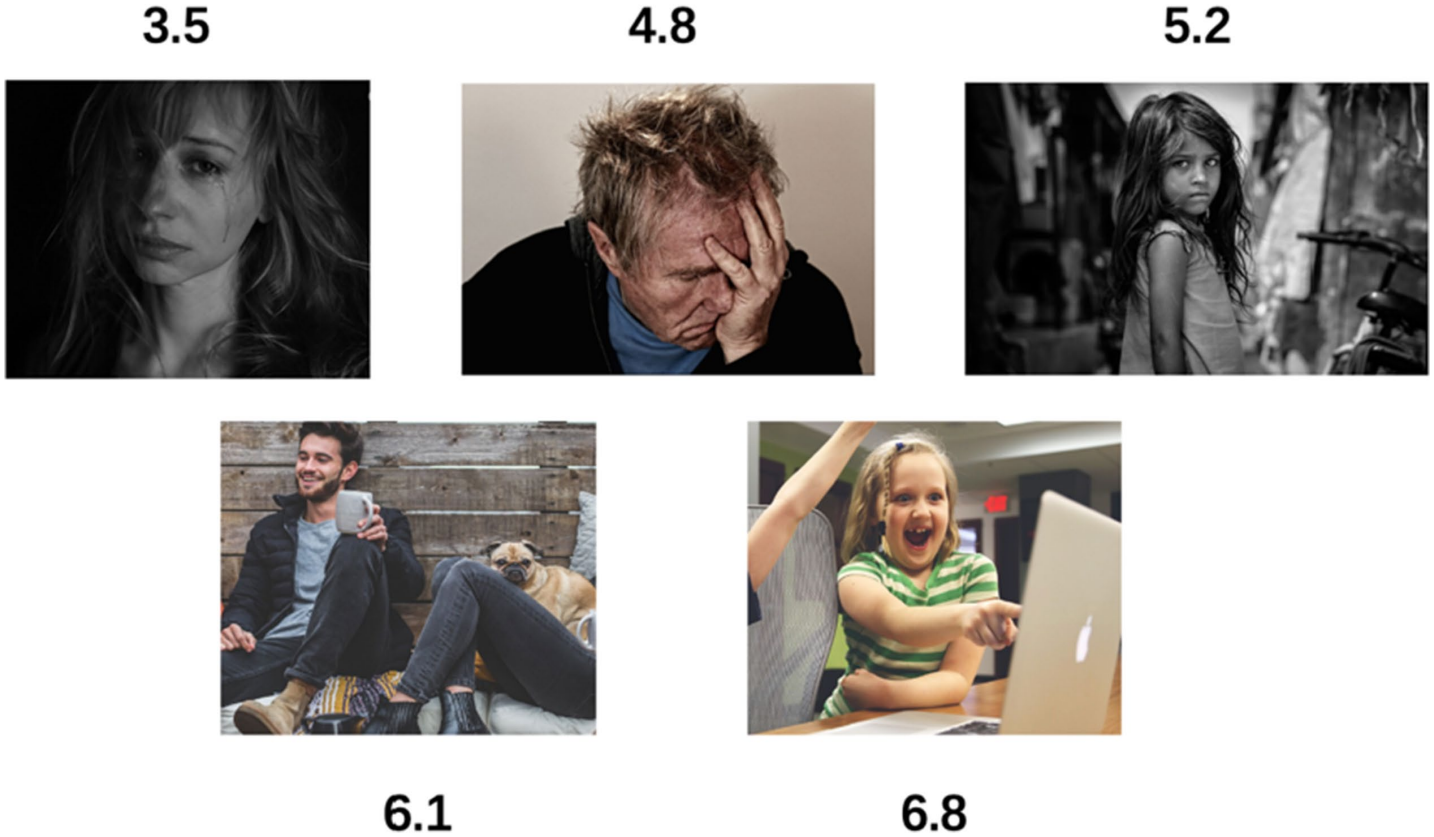
Emotion-V value correspondence. Specific values associated with are facial emotional values for each image.

Nevertheless, measuring emotion with precision can be a convoluted endeavor. Traditional perspectives tend to treat emotions as discrete words, such as anger, disgust, fear, happiness, sadness, and surprise (38). However, such a categorical format is not conducive to our quantitative analysis of a large number of films. In contrast, the VAD model of emotion describes emotions using three numerical dimensions (39): Valence (V), which measures the positivity or pleasantness of an emotion, ranging from negative to positive; Arousal (A), which measures the agitation level of the individual, ranging from non-active/calm to agitated/ready to act; and Dominance (D), which measures the level of control an individual feels over the situation, ranging from submissive/non-control to dominant/in-control. The valence dimension, which quantifies the positivity or pleasantness of an emotion on a spectrum from negative to positive, aligns seamlessly with our goal of capturing the emotional resonance and impact of religion-related films (23). As artistic works deeply intertwined with spiritual and existential themes, the ability to measure the degree of positive or negative emotional responses evoked by these films holds profound implications (40). By quantifying the valence values across our sample of religion-related and general acclaimed films, we can discern potential divergences in emotional trajectories, shedding light on how the portrayal of religious concepts may elicit distinct emotional experiences. Furthermore, the numerical nature of the valence metric facilitates rigorous quantitative analyses, enabling us to draw statistically grounded inferences and comparisons (41). While the arousal and dominance dimensions offer valuable complementary insights, the valence dimension emerges as the most germane and interpretable for our specific inquiry into the emotional landscape of religion-related cinema (42). For example, in Figure 4, results show that the valence value for the majority of scenes fluctuated between 3 and 7, with a value of 3.5 suggesting a very low emotional state, 5 representing a neutral emotional state, and 6.5 indicating a very happy emotional state.

## 3 Results

### 3.1 Descriptive results of 25 religion-related films

The overall results of the 25 religion-related films are depicted in Table 1. It presents a collection of these films, categorized by their respective religious affiliations, along with their corresponding emotional valence values and standard deviations (SD).

**Table 1:** Descriptive information of 25 religion-related films.

No.	Names	Religions	Values	SD
1	12 Years a Slave	Christianity	5.77	0.29
2	The Grapes of Wrath	Christianity	5.95	0.31
3	Schindler’s List	Christianity	5.41	0.53
4	Apocalypse Now	Others	5.78	0.27
5	Lady Bird	Christianity	5.69	0.35
6	There Will Be Blood	Christianity	5.69	0.32
7	Sita Sings the Blues	Buddhism	6.22	0.35
8	Spotlight	Christianity	5.5	0.41
9	Timbuktu	Islam	5.83	0.31
10	The Rider	Christianity	5.62	0.38
11	Moolaadé	Christianity	5.8	0.26
12	Atanarjuat: The Fast Runner	Others	5.74	0.39
13	Son of Saul	Buddhism	5.23	0.45
14	Ida	Christianity	5.29	0.6
15	Hard to Be a God	Christianity	5.54	0.33
16	Minari	Others	5.65	0.4
17	Wild Strawberries	Christianity	5.66	0.55
18	The Seventh Seal	Christianity	5.6	0.57
19	Maria Full of Grace	Christianity	5.49	0.5
20	The Wicker Man	Christianity	5.69	0.39
21	Divine Love	Christianity	5.88	0.26
22	The Master	Christianity	5.43	0.52
23	The Wild Pear Tree	Islam	5.72	0.36
24	Black Narcissus	Christianity	5.59	0.59
25	The Tree of Life	Christianity	5.63	0.42

The attribution of a film’s religious affiliation demands careful consideration, as individuals hold varying beliefs and employ distinct frameworks to comprehend the world. Given the diverse array of religions and belief systems globally, accurately categorizing a film’s affiliation proves challenging. Consequently, three independent researchers (coders) will delineate a film’s religious association by independently scrutinizing its primary plot (PP), the fundamental emotions it seeks to convey (FE), while considering the contextual backdrop (CB). Thus, inter-coder agreement was assessed using the Kappa statistic (0.83), and discrepancies were resolved through joint review.

First of all, Christianity emerges as the most prevalent religion depicted in the films, with the majority (18 out of 25) exploring Christian themes. The emotional valence values of the Christian-related films range from 5.29 to 5.95, with a mean value of approximately 5.62 and a standard deviation ranging from 0.26 to 0.6. These films demonstrate a relatively narrow range of emotional valence values within the context of Christianity, indicating a consistent portrayal of moderately positive emotional experiences. Besides, Buddhism is represented by two films in the table, namely “Sita Sings the Blues” and “Son of Saul,” with emotional valence values of 6.22 and 5.23, respectively. Compared to Christianity, the emotional spectrum depicted in these two films is more extensive, oscillating between profound melancholy and sheer elation, suggesting a more diverse representation of emotional experiences within Buddhist-related films. Further, Islam is portrayed in two films, “Timbuktu” and “The Wild Pear Tree,” with emotional valence values of 5.83 and 5.72, respectively. These films, on the whole, exhibit a prevailing sense of elevated positive emotional intensity, comparable to the Christian-related films but with slightly higher valence values.

While Christianity dominates the selection, the emotional valence values within the Christian-related films exhibit a relatively narrow range. In contrast, the films representing Islam, Buddhism, and others demonstrate a broader emotional spectrum within their respective traditions. These findings provide insights into the emotional landscapes depicted in religion-related films and indicate potential variations in emotional portrayal across different religious traditions, warranting further investigation.

### 3.2 Emotional patterns of religion-related film

To visualize and analyze emotional progression in religion-related films, we constructed emotion curves using data visualization techniques. The horizontal axis represents normalized film progression (percentage) for cross-film comparison, while the vertical axis reflects character emotion valence (V). Scenes without characters have a valence of zero and are excluded. For scenes with multiple characters, the average valence is used.

**Figure 5:**
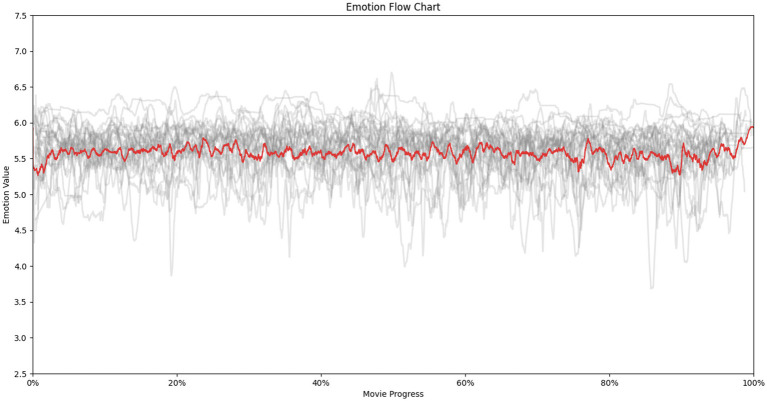
Emotional curve for all 25 religion-related films.

To manage the high data volume (tens of thousands of points per film), we employed smoothing techniques with a 100-point sliding window. Each gray line represents an individual film’s emotional trajectory, while the red line depicts the most frequent V value at each progression point (x-axis). This red line signifies the points of greatest convergence across all films, visually portraying shared emotional trends within the genre (Figure 5).

Figure 5 reveals a distinct emotional journey. Initially, valence exhibits a gradual rise from a low baseline. Around 3% of the film’s duration, a noticeable threshold is reached, indicating heightened audience engagement. Subsequently, between 3 and 75%, emotional valence fluctuates but maintains a level of stability, suggesting sustained emotional connection.

From 75 to 90% of film progression, valence continues to fluctuate with increasing amplitude, reflecting potential plot developments or moments of heightened emotional impact. Finally, the last segment (90–100%) witnesses a significant upswing in valence, reaching its peak. This suggests a climactic culmination of emotional engagement, possibly associated with the film’s climax or resolution.

The emotional curve analysis reveals a distinctive emotional trajectory for religion-related films. This trajectory is characterized by an initial rise in emotional valence, followed by a period of sustained fluctuation with increasing amplitude, culminating in a peak at the film’s conclusion. However, the generalizability of these findings is limited by the sample size of 25 films. While a common emotional pattern emerges within this specific dataset, it remains unclear whether this pattern is unique to religion-related films or reflects broader cinematic trends. To address this limitation and enhance the robustness of our conclusions, a comparative analysis employing a larger and more diverse sample of films is necessary.

**Figure 6:**
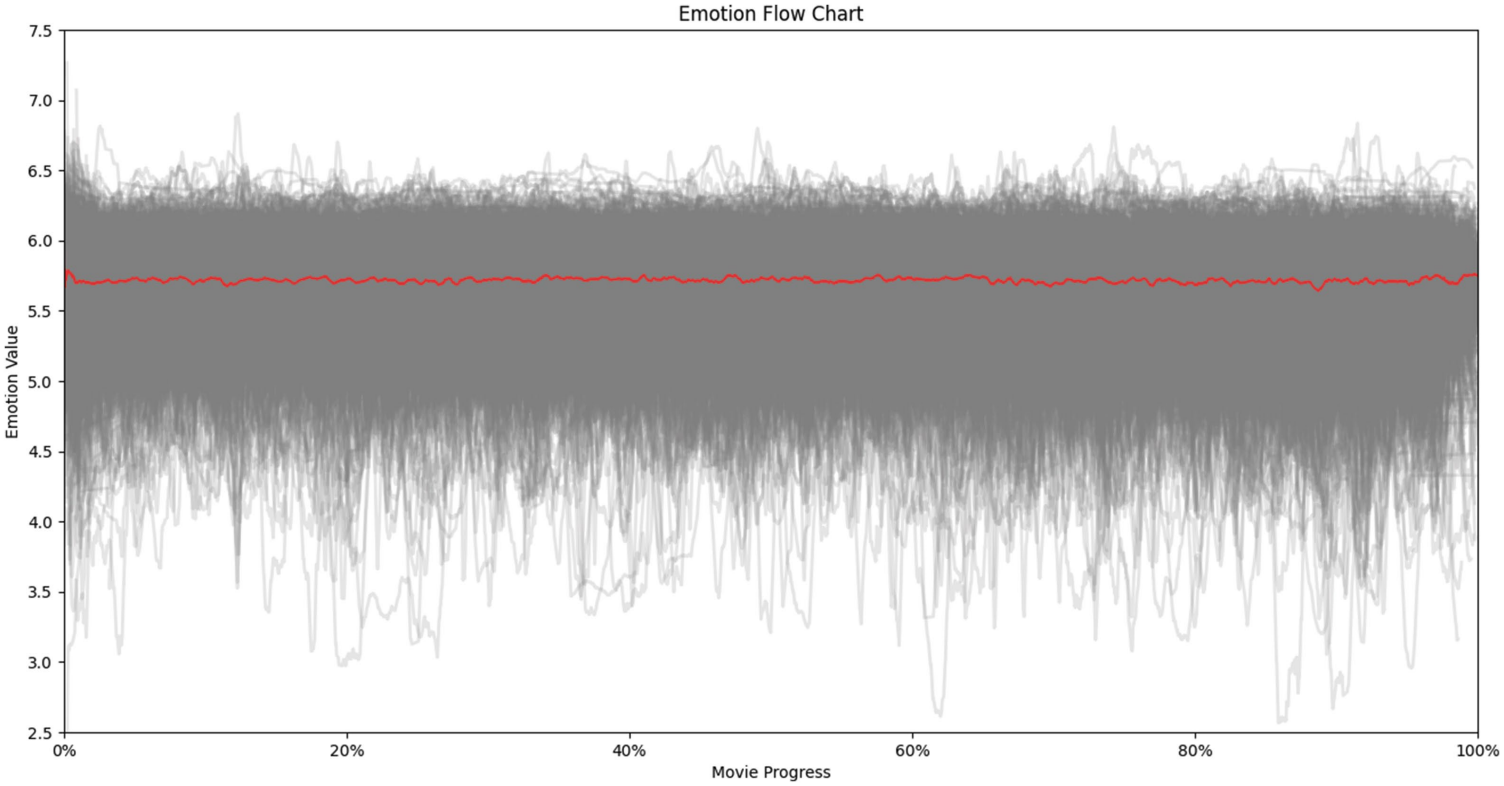
Emotional curve for all 2054 great films.

We implemented the same data visualization methodology on a substantially larger sample of 2,054 critically acclaimed films from various genres. The resulting emotional curve (Figure 6) exhibits a markedly different pattern. In contrast to the distinct trajectory observed in religion-related films, the emotional curve for the broader film sample appears near linear, lacking a well-defined pattern. This suggests a potential divergence in emotional expression trajectories across different film genres.

**Figure 7:**
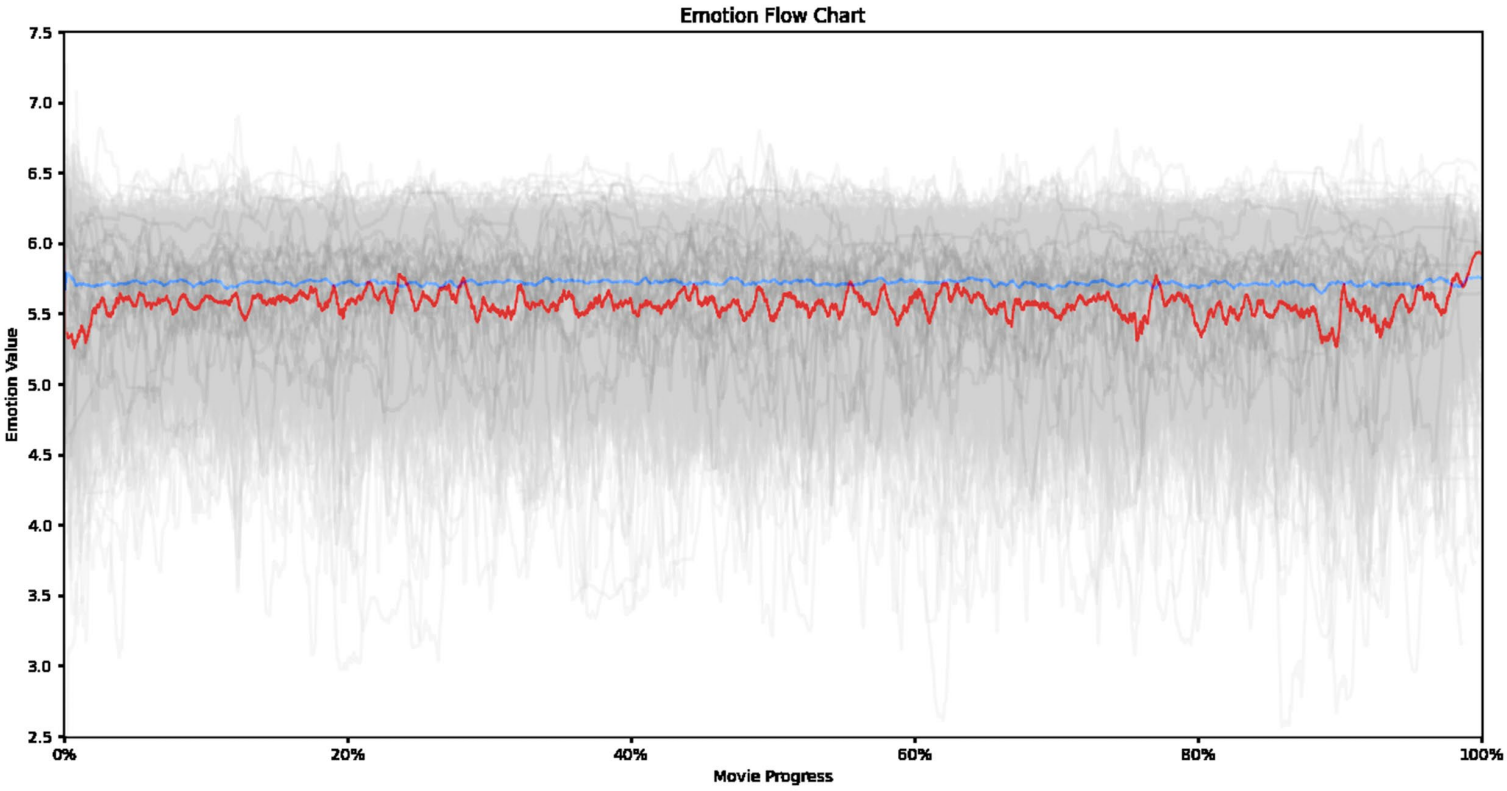
Emotional curve for both film datasets.

To further emphasize the contrast, we overlaid and resized both graphs with adjusted saturation and contrast for improved visual clarity (Figure 7). The red curve represents the 25 religion-related films, while the blue curve depicts the 2,054 critically acclaimed films. As illustrated in Figure 7, emotional expression patterns reveals a distinct characteristic for religion-related films compared to the broader landscape of acclaimed films.

## 4 Discussion

This study pioneers an investigation into the emotional landscapes of religion-related films and their potential influence on viewers through a cinematherapy lens. We employ computational techniques to map the emotional trajectories at pivotal narrative moments. By comparing these trajectories to a diverse sample of critically acclaimed films, we aim to illuminate the unique emotional dynamics that characterize religion-related films and their subsequent impact on religious narrative dissemination within therapeutic contexts.

Building upon the computational approach to uncover subtle, often unconscious communication in films (43), we analyze emotional trajectories in religion-related films. Our illustration of these emotional trajectories allows us to not only interpret the emotional tone at any given moment but also to trace its evolution throughout the film’s entirety. This ability enables us to uncover recurring emotional patterns within films from the same religious tradition or note significant deviations when comparing different traditions, objectively illuminating the specific emotional mechanisms underlying cinematherapy’s effectiveness.

Through this lens, we decipher the unique emotional trajectory of religion-related films, considering the influences of culture, belief systems, and religious tenets. First of all, compared to the broader landscape of acclaimed films (represented by the average curve of 2,054 films), religion-related films exhibit a consistently lower average emotional valence displayed by the characters throughout the narrative. This suggests that the portrayal of religious themes and values may emphasize a subtler range of emotions compared to the broader spectrum typically found in acclaimed cinema.

This muted emotional expression might be a deliberate choice by filmmakers. Religious narratives often grapple with profound questions about life, purpose, and morality (44). By presenting characters who navigate these complexities with a range of subtler emotions – contemplation, hope, quiet struggle – these films may create space for viewers to engage with the narrative on a more personal level (33). The lower intensity allows viewers to project their own emotional experiences onto the characters’ journeys, fostering introspection and potentially even inspiring personal growth (22).

This possibility aligns perfectly with the therapeutic potential of religion-related films within the cinematherapy framework. Unlike high-arousal films that might overwhelm viewers, the subtler emotional expression in religion-related films creates a safe space for self-reflection (4). Viewers can contemplate the characters’ journeys and underlying themes without feeling bombarded by intense emotions (4). This emotional safety zone may be particularly effective for therapeutic purposes, allowing viewers to engage with their own struggles and explore their values in the face of complex religious ideas (33).

Another notable observation is the synchronization between the shifts in the emotional curve of religion-related films and the patterns of spiritual transformation often accentuated in religious doctrines. Due to the uneven distribution of religious representation in our sample (18 out of 25 films being Christian), discussion focused on Christian doctrine and spirituality to ensure a more robust analysis. To specify, Christianity’s narratives revolve around themes of redemption, salvation, and spiritual growth, often portrayed through story arcs that mirror journeys of faith and quests for divine connection (45–47). Our observed emotional curves strikingly align with these narrative structures (48, 49). They depict the archetypal progression from spiritual emptiness to the pinnacle of divine encounter, each phase resonating deeply with the viewer and fostering spiritual connectivity (50–52).

To illustrate, the initial low point in the emotional trajectory corresponds to Christian doctrine’s portrayal of the human condition—separated from God and in need of redemption (Romans 3:23). This aligns with the concept of original sin and the need for redemption, which forms a foundational aspect of Christian theology (53). As the narrative unfolds, the fluctuating emotional values signify the trials and tribulations of the spiritual journey, echoing the quest for reconciliation with the divine (43, 51, 54). This journey is marked by the quest for spiritual growth, reconciliation, and a deepening relationship with God (11, 12, 55). It is within this narrative arc that characters grapple with their faith, facing moments of crisis and personal struggle (51, 56). Last, the c signifies the climactic moment of spiritual transformation and revelation, reflecting the Christian belief in divine intervention, grace, and the experience of a personal encounter with God (45–47). Such moments of revelation evoke heightened emotional responses, including feelings of awe, inspiration, and a profound connection with the divine (Romans 3:23; Ephesians 2:8–9).

“The Tree of Life,” directed by Terrence Malick, serves as a compelling illustration that highlights the emotional trend observed in Christian-related films. This experimental drama intertwines the story of a Texas family in the 1950s with contemplative reflections on the nature of existence, creation, and spirituality. To specify, this film introduces the O’Brien family at the beginning, a young family brimming with the joy and innocence of early childhood. This initial emotional state sets the baseline for the film. As the narrative progresses, mirroring the family’s growth and their expanding world view, the emotional tenor gradually intensifies. A pivotal moment early in the film throws the family, and the audience by extension, into a profound state of grief and existential questioning. This event marks a significant shift in the emotional landscape, with viewers deeply invested in the characters’ struggle with loss. The film maintains a relatively stable yet dynamic emotional state throughout the middle section. This phase explores the complexities of family life, weaving themes of love, loss, forgiveness, and the search for meaning. The consistent emotional sentiment reflects the family’s unwavering faith and the Christian film’s spiritual underpinnings. However, as the film nears its conclusion, the emotional intensity escalates, mirroring the protagonist’s deepening introspection, existential struggles, and attempts at reconciliation within their faith. The narrative captures the characters’ evolving emotional states and spiritual growth, translating their internal conflicts into a powerful audience experience. Finally, the film reaches its emotional and narrative peak in the closing moments, culminating in themes of divine grace, redemption, and the interconnectedness of all existence. This emotional crescendo encapsulates the essence of faith and the characters’ conviction in a higher power. Similar observations can be found in films like “Hard to Be a God” and “Divine Love,” suggesting a potential link between Christian themes and this unique emotional arc.

“The Tree of Life’s” narrative pattern fosters a unique viewer engagement that surpasses conventional storytelling. It utilizes film as a reflective canvas, enabling viewers to see their own spiritual and emotional struggles reflected in the characters’ journeys (52). This reflective process is not only therapeutic but may also serve as a catalyst for personal transformation, as the film’s narrative progression encourages viewers to contemplate their place in the world, their relationships, and their beliefs (46).

This study acknowledges the limitations inherent in relying solely on quantitative analysis for understanding the emotional impact of religion-related films. While quantitative analysis offers valuable insights into the emotional impact of religion-related films, it inherently struggles to capture the full spectrum of human emotional experience, particularly the subtle nuances that reside within that spectrum (57). Capturing the full spectrum of human emotions, particularly the subtle emotional cues that can hold profound meaning for individuals, remains a challenge (58). Moreover, while our study strived to account for cultural factors through these methodological approaches, we acknowledge that the influence of culture on film interpretation is complex and multifaceted. Further research is needed to explore the nuances of cultural factors in the analysis of religion-themed films across diverse cultural contexts (59). Additionally, attributing therapeutic benefits solely to film exposure requires further investigation in controlled settings (7). While cinema undoubtedly plays a significant role in shaping our psychological and emotional well-being, the therapeutic effects of these films require careful consideration of individual differences, religious backgrounds, and potential psychological complexities (60).

## 5 Conclusion

This study investigated how religion-related films influence viewers through a “cinematherapy” lens. We used facial recognition to map emotional patterns in these films, contrasting them with a wider range of acclaimed movies. Religion-related films unveiled a distinct emotional journey. Compared to the broader film spectrum, they exhibited a subtler range of emotions, potentially fostering a contemplative space for viewers. This aligns with religious themes that often grapple with profound existential questions. The emotional curve mirrored spiritual journeys depicted in these films, starting with a low point symbolizing separation from the divine and culminating in a peak representing spiritual transformation. These findings suggest religion-related films hold promise for cinematherapy. The subtle emotional expression creates a safe space for self-reflection, allowing viewers to connect with the characters’ struggles and explore their own values in the face of complex religious ideas. This emotional engagement may contribute to therapeutic goals like introspection and personal growth. Unveiling the unique emotional power of religion-related films, this study paves the way for further research on their potential therapeutic applications.

## Data availability statement

The raw data supporting the conclusions of this article will be made available by the authors, without undue reservation.

## Author contributions

BX: Conceptualization, Writing – original draft. ZW: Formal analysis, Methodology, Writing – review & editing. YL: Writing – review & editing, Investigation. YS: Supervision, Writing – review & editing.
